# Endemic Circulation and Genetic Characterization of Foot‐and‐Mouth Disease Virus in Buffalo Populations of Bangladesh

**DOI:** 10.1155/tbed/3107202

**Published:** 2026-09-12

**Authors:** Abdullah Mohammad Asif, Tajul Islam Mamun, Md. Rokibul Hasan Shanto, Md. Khademul Islam, Md. Mashfiq Hossain Khadem, Uttama Acharjee, Md. Shazzadul Kabir, Md. Shakil Mahmud Supto, Asikur Rahman, Syed Muktadir Al Sium, Amam Zonaed Siddiki, Md Bashir Uddin, Syed Sayeem Uddin Ahmed

**Affiliations:** ^1^ Department of Epidemiology and Public Health, Sylhet Agricultural University, Sylhet 3100, Bangladesh, sau.ac.bd; ^2^ Department of Medicine, Sylhet Agricultural University, Sylhet 3100, Bangladesh, sau.ac.bd; ^3^ Bangladesh Council of Scientific and Industrial Research, Dhaka 1205, Bangladesh, bcsir.gov.bd; ^4^ Department of Pathology and Parasitology, Chattogram Veterinary and Animal Sciences University, Chattogram 4202, Bangladesh, cvasu.ac.bd; ^5^ Faculty of Veterinary Medicine and Animal Science, Habiganj Agricultural University, Habiganj 3300, Bangladesh, hau.ac.bd

**Keywords:** Bangladesh, buffalo, foot-and-mouth disease virus, genetic diversity, molecular epidemiology, VP1 gene

## Abstract

Foot‐and‐mouth disease (FMD) virus (FMDV) is endemic in Bangladesh, causing severe economic losses in the livestock sector. While it primarily affects cattle, buffaloes (*Bubalus bubalis*) remain highly susceptible. Therefore, this study aimed to determine the prevalence and molecular characteristics of FMDV in buffaloes across three districts (Sylhet, Rajshahi, and Noakhali) of Bangladesh from January to June 2024. In a cross‐sectional study, a total of 622 nasal swabs from 67 herds were collected and tested for FMDV RNA using reverse transcription polymerase chain reaction (RT‐PCR). Overall, 255 samples were positive, resulting in an individual‐level prevalence of 41.0%(255/622), while 88.1% (59/67) of herds were FMDV‐positive. Animal‐level prevalence was highest in Sylhet (47.1%), followed by Noakhali (38.9%) and Rajshahi (37.1%). To further characterize circulating strains, eight representative RT‐PCR‐positive samples were sequenced, revealing the co‐circulation of serotypes O (*n* = 5) and Asia‐1 (*n* = 3). Phylogenetic analysis showed that the isolates belonged to the ME‐SA/Ind2001e lineage of the serotype O and the Asia‐1 Group V lineage, clustering with contemporary strains from Bangladesh and neighboring countries, suggesting possible intra‐ and transboundary transmission. Pairwise genetic distance evaluation revealed high regional similarity, while Mantel tests indicated significant associations between genetic, geographic, and temporal distances. Comparative genomic analysis revealed largely conserved genomic regions, whereas VP1 analysis indicated that purifying selection predominated across both serotypes, with serotype O exhibiting greater genetic diversity (*π* = 0.11642) than Asia‐1 (*π* = 0.05258), suggesting localized antigenic variability and possible immune‐mediated viral evolution. These findings highlight the need for strengthened surveillance, improved biosecurity, and integrated vaccination strategies to enhance FMD control and reduce economic losses in Bangladesh.

## 1. Introduction

Foot‐and‐mouth disease (FMD) is a highly contagious transboundary viral disease of cloven‐hoofed animals, including cattle, buffalo, sheep, goats, and pigs, and is endemic across Africa and Asia, including Bangladesh [1]. It causes substantial economic losses due to reduced productivity, trade restrictions, and high morbidity, which may reach up to 100% [2–5]. In young animals, mortality may occur due to myocarditis, while in adults, the disease primarily results in decreased milk yield, weight loss, and reproductive problems [6]. Although extensive research has been conducted on FMD in cattle, buffalo (*Bubalus bubalis*) remains comparatively understudied in Bangladesh. Buffaloes are an important component of the livestock sector and contribute significantly to rural livelihoods and food security. Recent studies suggest that buffalo may participate in the maintenance and circulation of FMD virus (FMDV) as potential reservoir hosts [1, 7, 8].

The causative agent, FMDV, is an antigenically diverse RNA virus of the genus *Apthovirus* (family Picornaviridae), comprising seven serotypes: O, A, C, Asia‐1, SAT 1, SAT 2, and SAT 3, reported worldwide [3, 9, 10]. Among these, serotypes O, A, and Asia‐1 are predominantly reported in South Asia, including Bangladesh, where unrestricted animal movement and porous international borders facilitate virus transmission [2, 3].

Buffaloes are multipurpose animals that provide milk, meat, draught power, and income for farming households. Bangladesh possesses a substantial buffalo population, particularly in coastal, wetland, and river basin ecosystems [11]. Globally, the buffalo populations are substantial, with the majority found in Asia. In Bangladesh, a considerable number of buffaloes are reared, mainly on grass and agricultural by‐products [12]. Despite their economic significance, buffaloes remain underrepresented in national livestock health research. Infectious diseases, including FMD, cause considerable losses through reduced growth, decreased milk and meat production, and impaired work output [5, 7, 13, 14]. Studies have also shown a higher seroprevalence of FMDV antibodies in buffalo compared to that in cattle [15], with morbidity in buffalo reported at 23.3% [16].

Vaccination remains the primary control strategy against FMD in Bangladesh. However, vaccine effectiveness may be compromised by continual viral evolution and antigenic divergence among circulating strains [17]. Molecular surveillance using reverse transcription polymerase chain reaction (RT‐PCR), sequencing, and phylogenetic analyses has become increasingly important for identifying circulating strains and informing vaccine selection [3, 18, 19].

Recent molecular epidemiological studies conducted in Bangladesh have predominantly characterized FMDV strains from cattle populations, identifying the circulation of serotypes O, A, and Asia‐1 and documenting the emergence of novel viral lineages [2, 3, 20, 21]. Although these studies have substantially improved understanding of FMDV evolution in cattle, comparable molecular data for buffalo remain scarce. Consequently, little is known about the prevalence, genetic diversity, phylogenetic relationships, and evolutionary dynamics of FMDV circulating specifically within the buffalo populations in Bangladesh. Therefore, the present study aimed to estimate the prevalence of FMDV infection and molecularly characterize the circulating FMDV strains among buffalo populations in selected regions of Bangladesh. Understanding the epidemiological and evolutionary characteristics of buffalo‐derived FMDV isolates will contribute to evidence‐based surveillance and control programs and support the development of effective FMD management strategies.

## 2. Materials and Methods

### 2.1. Ethical Approval

All samples were collected by expert veterinarians considering ethical standards and animal welfare issues. Informed verbal or written consent was obtained from the animal owners before sample collection in accordance with approved ethical guidelines. All procedures were conducted in accordance with the ethical standards approved by the Animal Experimentation Ethics Committee of Sylhet Agricultural University (Protocol # AUP2023029). This study was conducted in compliance with the ARRIVE guidelines.

### 2.2. Study Design

This study was conducted in three purposively selected districts of Bangladesh: Sylhet (upazila: Fenchuganj), Rajshahi (upazila: Godagari), and Noakhali (upazila: Companiganj) based on high buffalo density, history of recurrent FMD outbreaks, and representation of geographical regions of Bangladesh (haor/wetland, northwest plain land, and coastal belt) [3, 22]. The target population included buffalo of all ages, regardless of sex. Herds and individual buffaloes were selected from official livestock office records, with the support of the Bangladesh Livestock Research Institute (BLRI). Animals were randomly selected at the time of visit. A cross‐sectional study was conducted from January 2024 to June 2024, a period associated with increased FMD occurrence due to seasonal and post‐monsoon factors in Bangladesh.

### 2.3. Sample Collection

Samples were collected from buffaloes in both symptomatic and asymptomatic (apparently healthy) herds to capture the full spectrum of infection status. During field visits, animals were clinically examined and their health status was recorded using a structured questionnaire before the sampling.

**Figure 1:**
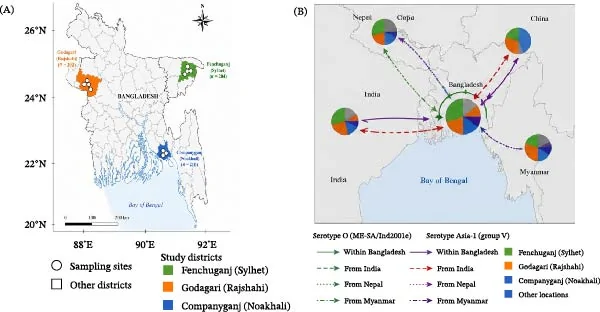
Geographical location of the selected study area. (A) Study area and sampling locations and (B) phylogeographic reconstruction (discrete trait analysis). Arrow indicate the most probable migration routes.

An initial field survey was conducted to assess the disease occurrence and facilitate sample collection in the study areas. A two‐stage sampling approach was employed, where study areas were purposively selected based on predefined criteria, and buffaloes were subsequently selected using simple random sampling during field visits irrespective of clinical status. To ensure adequate statistical power for prevalence estimation, the sample size was calculated using the standard formula: *n* = (*Z* ^2^ × *P* × (1−*P*))/*d* ^2^, where *Z* = 1.96 (95% confidence level), *p* = 0.5 (expected prevalence due to limited prior buffalo‐specific data), and *d* = 0.05 (precision). The calculated minimum sample size was exceeded to improve precision and account for clustering at the herd level. Therefore, a total of 622 nasal swab samples were collected from 67 buffalo herds (herd size: 10–66) across three upazilas, comprising 204 samples from Fenchuganj, 202 from Godagari, and 216 from Companiganj (Figure 1A). Samples were placed in 3 mL viral transport medium (VTM), labeled with identification codes, and transported in an ice box at 4–8°C to the Department of Epidemiology and Public Health Laboratory, where they were stored at –40°C until processing.

#### 2.3.1. Extraction and Detection of FMDV by RT‐PCR

Viral nucleic acid (RNA) was extracted from collected nasal swab samples using the AddPrep Viral Nucleic Acid Extraction Kit (Korea) following the manufacturer’s protocol and immediately processed. The remaining samples were stored at −80°C for further use. For the detection of FMDV, all extracted RNAs were subjected to conventional RT‐PCR using 10 pmol of each primer. A MiniAmp Thermal Cycler (Thermo Fisher Scientific) was used to perform this RT‐PCR. Primer list is included in Table S1. Briefly, the RT‐PCR targeted the FMDV polyprotein gene and was performed under standard cycling conditions, including cDNA synthesis at 55°C for 10 min, initial denaturation at 95°C for 5 min, followed by 45 cycles of denaturation at 95°C for 30 s, annealing at 57°C for 45 s, and extension at 72°C for 45 s, with a final extension step. Safe‐Red Gel was used to visualize the corresponding amplification site of the polyprotein gene on a 1.5% agarose gel (Agarose LE; BC0015, 100 g) in gel electrophoresis. E‐Gel Power Snap (Invitrogen) was used for gel documentation. The expected amplicon sizes for the FMDV‐positive sample using both NPP and RF2 primers (Table S1) were 494 and 215 bp, respectively.

For RT‐PCR assay validation, a previously confirmed field isolate of FMDV obtained from cattle was used as the primary positive control. This isolate was molecularly characterized and served as a reliable source of intact viral RNA. In addition, the Bangla FMD Vac inactivated vaccine was included as a supplementary reference control to verify primer reactivity against vaccine strain antigens. All positive control amplifications were further validated through sequencing to confirm the specificity of the RT‐PCR assay.

### 2.4. Statistical Analysis

For this study, individual‐level prevalence was defined as the proportion of buffaloes that tested positive for FMDV by RT‐PCR using nasal swabs, whereas herd‐level prevalence was defined as the proportion of herds with at least one buffalo testing positive within the group. The prevalence of infection was calculated at both animal and herd levels. Animal‐level prevalence was determined as the proportion of positive animals out of the total animals tested, expressed as a percentage. Herd‐level prevalence was calculated as the proportion of herds with at least one positive animal relative to the total herds sampled. Differences in animal‐level prevalence and herd‐level prevalence among study areas were assessed using Pearson’s chi‐square test. Statistical significance was considered at *p* < 0.05. The geographical distribution of positive herds was mapped in ArcMap 10.7.

### 2.5. Phylogenetic and Evolutionary Analysis

Eight representative FMDV‐positive samples (BD S1–S8) were sequenced based on RT‐PCR amplification quality (clear band intensity indicating sufficient viral RNA) and geographic distribution across the three study upazilas (Fenchuganj, Godagari, and Companiganj) to capture regional variation in circulating strains. However, the selection strategy did not explicitly incorporate herd‐level representation, epidemiological clustering, or temporal outbreak dynamics, which may introduce a potential sampling bias. The obtained nucleotide sequences were initially analyzed using BLAST (https://blast.ncbi.nlm.nih.gov/Blast.cgi) to confirm the sequence identity and to identify closely related strains available in the GenBank database. Based on sequence similarity, geographic distribution, and sequence completeness, appropriate reference sequences for both partial‐genome regions and whole‐genome analyses were retrieved from the National Center for Biotechnology Information (NCBI) GenBank database (https://www.ncbi.nlm.nih.gov/genbank/) and incorporated into subsequent evolutionary analyses. The generated sequences have been deposited in the NCBI GenBank database, and the corresponding accession numbers are provided in the Data Availability section.

Multiple sequence alignment for both partial and whole‐genome datasets was performed using MAFFT v7 with default parameters, ensuring the use of homologous genomic regions for serotypes O and Asia‐1 [23]. Partial sequences were trimmed at both ends using block mapping and gathering with entropy (BMGI) to remove low‐quality and non‐overlapping regions before phylogenetic reconstruction [24]. The most suitable nucleotide substitution model was selected based on the lowest Bayesian information criterion (BIC) score and Akaike information criterion (AICc) value in the MEGA11 program [25]. Phylogenetic relationships were subsequently inferred using the maximum likelihood method implemented in RAxML v8 under the general time reversible model with gamma‐distributed rate variation among sites (GTR + G), and node support was evaluated through bootstrap analysis with 1000 replicates [26–29]. The resulting trees were visualized and annotated using Interactive Tree of Life (iTOL v7), incorporating the geographic origins of isolates to facilitate interpretation of evolutionary relationships among circulating strains.

### 2.6. Genetic Distance Analysis

Pairwise genetic distances among FMDV whole‐genome sequences (WGS) were calculated using the Kimura 2‐parameter model in MEGA, which employs standard molecular evolution models to estimate genetic divergence between sequences. The resulting genetic distance matrix was used to assess the relationships between genetic divergence and epidemiological factors. Geographic distances were calculated from the sampling locations, and temporal distances were derived from sampling dates. Correlations between genetic and geographic, as well as genetic and temporal distances, were evaluated using the Mantel test with 999 permutations. The Mantel test was selected as it is appropriate for assessing correlations between non‐independent pairwise distance matrices. Linear regression analysis was further applied using the model: *Y* = *β*_0_ + *β*_1_ *X*, where *Y* represents genetic distance and *X* represents geographic or temporal distance between samples, to assess trends between variables. The assumptions of linear regression, including linearity and independence, were considered during the analysis. Regression lines were visualized, and correlation coefficients (*r*) were reported. All statistical analyses were performed in Python (v3.11) using the Pandas and SciPy libraries.

### 2.7. VP1 Amino Acid Variation and Selection Analysis

The VP1 coding region was extracted from the WGS generated in this study using a reference‐guided approach. Reference sequences AY304994.1 for serotype Asia‐1 and NC_004004.1 for serotype O were used to identify and extract the corresponding VP1 regions from the dataset. The extracted VP1 nucleotide sequences were aligned and translated into amino acid sequences. Amino acid variation, mutation positions, residue frequency, and GH loop regions were identified and analyzed using custom scripts implemented in Python (v3.11).

Selection pressure analysis of the VP1 gene was performed using codon‐based methods implemented in the HyPhy software package [30]. Fixed effects likelihood (FEL), fast unconstrained Bayesian approximation (FUBAR), mixed effects model of evolution (MEME), and single‐likelihood ancestor counting (SLAC) approaches were applied to estimate nonsynonymous and synonymous substitution patterns at individual codon sites [30–33].

### 2.8. Population Genetic Analysis of the VP1 Region

Population genetic and evolutionary analyses were subsequently performed using VP1 gene sequences of serotypes O and Asia‐1 due to their higher genetic variability and established role in FMDV antigenic and evolutionary characterization. Genetic diversity indices, including nucleotide diversity and haplotype diversity, were estimated using DnaSP v6 [34]. Neutrality tests, including Tajima’s *D* and Fu and Li’s statistics, were calculated to infer evolutionary dynamics within circulating populations. Distinct haplotypes were identified based on nucleotide variation, and a haplotype network was constructed using PopART to visualize genealogical relationships and mutational connections among isolates.

## 3. Results

### 3.1. Molecular Prevalence

A total of 622 nasal swab samples were collected from buffaloes across three Upazilas and screened for FMDV using the NPP and RF2 primer sets. Overall, 255 samples (41.00%) tested positive for FMDV. Fenchuganj showed the highest animal‐level prevalence (47.06%; 96/204; 95% CI: 40.05–54.15), followed by Companiganj (38.89%; 84/216; 95% CI: 32.35–45.74) and Godagari (37.13%; 75/202; 95% CI: 30.45–44.19) (Table 1). Chi‐square test indicated no statistically significant difference in animal‐level prevalence across the three Upazilas (*χ* ^2^ = 4.745, df = 2, *p* = 0.093). At the herd level, 59 out of 67 herds (88.06%; 95% CI: 77.82–94.70) were positive for FMDV. Herd‐level prevalence was highest in Companiganj (100%; 95% CI: 76.84–100.00), followed by Fenchuganj (88.00%; 22/25 herds; 95% CI: 68.78–97.45) and Godagari (82.14%; 23/28 herds; 95% CI: 63.11–93.94) (Table 1). Similarly, no significant difference was observed in herd‐level prevalence across Upazilas (*χ* ^2^ = 2.831, df = 2, *p* = 0.243). Due to small expected frequencies in some cells, Fisher’s exact test was also performed at herd‐level, which confirmed the lack of significant difference (*p* = 0.311).

**Table 1:** Animal and herd‐level prevalence of FMDV in buffalo.

Upazila	Animal‐level			Herd‐level		
	*N* (positive)	Prevalence (%) (95% CI)	*p*‐Value	*N* (positive)	Prevalence (%) (95% CI)	*p*‐Value
Fenchuganj	204 (96)	47.06 (40.05–54.15)	0.093	25 (22)	88.00 (68.78–97.45)	0.243
Godagari	202 (75)	37.13 (30.45–44.19)		28 (23)	82.14 (63.11–93.94)	
Companiganj	216 (84)	38.89 (32.35–45.74)		14 (14)	100 (76.84–100.00)	
Total	622 (255)	41.00 (37.10–44.98)	—	67 (59)	88.06 (77.82–94.70)	—

*Note: N* = total; *n* = positive.

### 3.2. Detection of FMDV Strain

To further characterize the circulating FMDV strains, sequencing analysis was performed. Eight representative RT‐PCR‐positive samples (BD S1–S8) were selected for genome sequencing using the Sanger method based on geographic distribution across the three Upazilas. BLAST analysis identified five sequences as FMDV serotype O and three as serotype Asia‐1 (Table S2), indicating the co‐circulation of multiple serotypes in the study regions. Although only eight samples were sequenced from 255 RT‐PCR‐positive samples, they were selected to represent different locations and high‐quality RNA. These data provide a preliminary insight into the circulating FMDV strains. However, the findings should be interpreted with caution due to the limited number of sequenced samples.

### 3.3. Phylogenetic and Evolutionary Analysis

**Figure 2:**
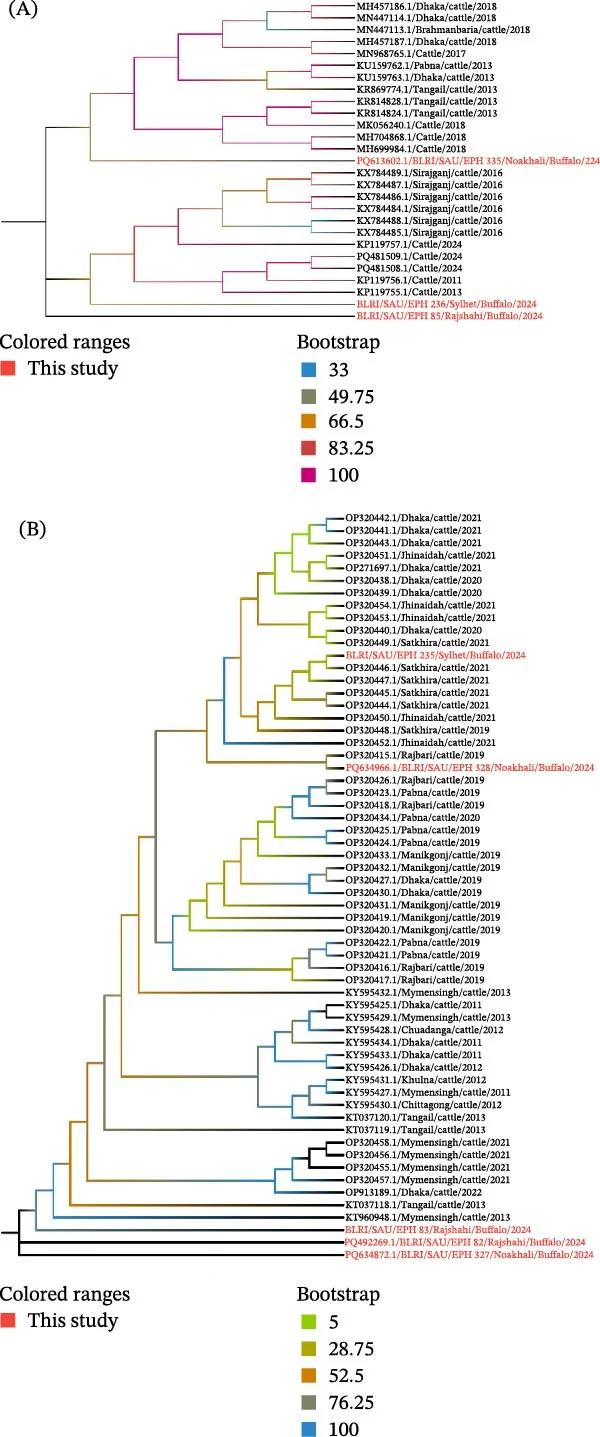
Phylogenetic tree of FMDV. Maximum likelihood phylogenetic tree based on partial nucleotide sequences of FMDV (A) serotype Asia‐1 and (B) serotype O circulating in Bangladesh.

To investigate the evolutionary relationships among the detected strains, phylogenetic analysis was conducted. Phytogeography analysis depicts Bangladesh as a central hub with inferred viral movement between regions and neighboring countries such as India, Nepal, and Myanmar, highlighting regional connectivity and transboundary spread (Figure 1B). Maximum likelihood phylogenetic analysis based on partial VP1 sequences demonstrated that the FMDV isolates generated in this study clustered within previously reported Bangladesh lineages for both serotypes O and Asia‐1. In the Bangladesh‐restricted phylogeny of serotype Asia‐1, the study isolates from Noakhali, Sylhet, and Rajshahi grouped within a single major clade together with recent Bangladesh reference sequences, supported by moderate to high bootstrap values (generally ≥70%) (Figure 2A). The sequences showed short branch lengths, indicating limited genetic divergence among the circulating Asia‐1 viruses. Similarly, in the Bangladesh‐based phylogenetic tree of serotype O, the study isolates clustered with previously reported Bangladesh strains (Figure 2B). Rajshahi and Noakhali isolates formed a well‐supported sub‐cluster, while the Sylhet isolate remained within the same lineage with moderate support. When analyzed together with global reference sequences, the Asia‐1 isolates from this study grouped within the established South Asian lineage, clustering with regional strains, including strains previously reported within the Asia‐1 group V lineage from East and Southeast Asia (Figure S1A). A similar pattern was observed for serotype O. The isolates belonged to the dominant South Asian lineage, Middle East–South Asia (ME–SA) topotype, and the Ind2001e lineage. The isolates clustered closely with strains reported from India and neighboring countries (Figure S1B). The maximum likelihood phylogenetic tree based on WGS showed well‐supported clustering within established FMDV lineages (Figure S2). These isolates clustered with geographically relevant South Asian strains within regional lineages. Due to the sequencing of only representative isolates, the lineage‐specific prevalence could not be determined across all samples.

### 3.4. Pairwise Genetic Distance and Regional Comparison of FMDV Sequences

**Figure 3:**
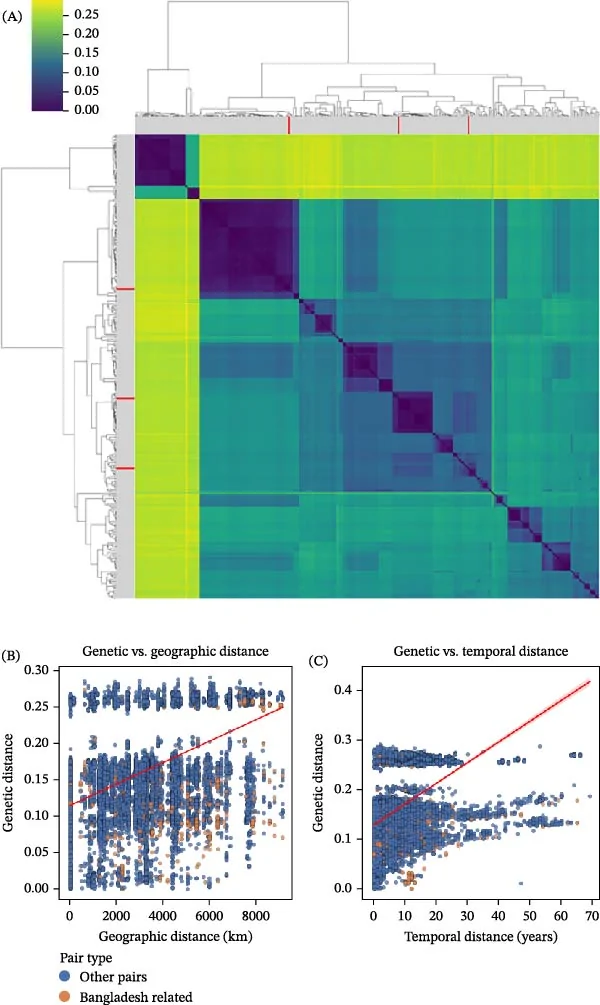
Genetic and geographic distances among FMDV whole‐genome sequences. (A) Hierarchical clustering heatmap of pairwise genetic distances among FMDV whole‐genome sequences (WGS). Colors indicate genetic distance, with darker shades representing higher similarity (lower genetic distance) and lighter shades indicating greater genetic divergence (higher genetic distance). The red line indicates the regression trend showing increasing genetic divergence with greater geographic distance (B), and temporal distance (C).

To further explore genetic relationships among the sequences, pairwise distance analysis was performed. Pairwise genetic distance analysis revealed clustering patterns among the analyzed sequences, consistent with the hierarchical clustering heatmap shown in Figure 3A. The study isolates PQ801122/Bangladesh/2024, PQ807430/Bangladesh/2024, and PQ807429/Bangladesh/2024 were grouped within regionally circulating lineages but showed variable genetic relatedness with sequences from other countries. PQ801122/Bangladesh/2024 showed the highest similarity with Bangladesh isolates, followed by Nepal, Saudi Arabia, China, and India, indicating regional evolutionary continuity. PQ807430/Bangladesh/2024 clustered closely with Indian isolates, showing minimal pairwise distances and suggesting strong genetic conservation within the South Asian cluster. In contrast, PQ807429/Bangladesh/2024 showed the highest similarity with Pakistan isolates, including near‐identical distances, indicating close phylogenetic relatedness. Overall, the clustering pattern reflects geographic structuring with genetic continuity among regional strains (File S1).

### 3.5. Regional Comparison of Genetic, Geographic, and Temporal Distances

Genetic and geographic distance analysis showed a moderate positive correlation between spatial separation and genetic divergence (Figure 3B; Table S3). The Bangladesh isolates followed the overall pattern and were distributed within intermediate genetic distance ranges. Similarly, a positive association between genetic and temporal distance was observed (Figure 3C), with isolates showing relatively lower genetic divergence over shorter time intervals.

### 3.6. VP1 Amino Acid Variability and Buffalo‐Specific Mutations in Serotype Asia‐1 and O FMDV

**Figure 4:**
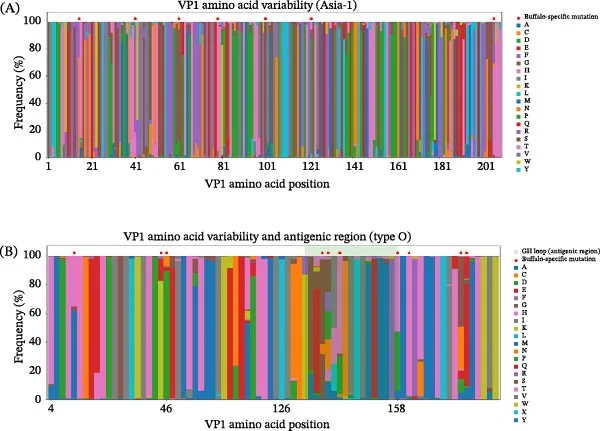
Amino acid variability in FMDV serotypes. (A) Amino acid frequency distribution across VP1 positions of FMDV serotype Asia‐1 showing overall conservation with selected buffalo‐specific mutations indicated by red markers. (B) Amino acid frequency distribution across VP1 positions of FMDV serotype O, highlighting buffalo‐specific mutations (red markers) and increased variability within the GH loop antigenic region.

Analysis of VP1 amino acid variability among serotype Asia‐1 and O FMDV sequences showed that most amino acid positions were highly conserved, indicating structural and functional constraints within the VP1 protein (Figure 4; Files S2 and S3). Despite this conservation, distinct buffalo‐specific amino acid substitutions were identified in the study sequences compared with the consensus profiles. In the serotype Asia‐1 sequence (PQ807429/Bangladesh/2024), substitutions were observed at positions 15 (C→R), 41 (R→H), 61 (G→N), 79 (T→I), 101 (S→G), 122 (P→S), and 206 (H→R) (Table 2). Similarly, in serotype O sequences, mutations were detected at positions 13 (A→T), 45 (K→Q), 46 (D→N), 139 (S→G), 140 (S→A), 142 (T→P), 158 (T→P), 171 (T→A), 197 (S→D), and 198 (E→Q) (Table 2). Several serotype O substitutions were located within or near the GH loop region (~135–160), an antigenically important region of VP1 (Table 3). Overall, these findings suggest localized variability within an otherwise conserved VP1 region in buffalo‐derived FMDV sequences.

**Table 2:** Buffalo‐specific VP1 amino acid substitutions identified in FMDV serotype Asia‐1 and serotype O sequences.

Strain	Sequence	Position	Majority AA	Buffalo AA
Asia‐1	PQ807429/Bangladesh/2024	15	Cysteine	Arginine
	PQ807429/Bangladesh/2024	41	Arginine	Histidine
	PQ807429/Bangladesh/2024	61	Glycine	Asparagine
	PQ807429/Bangladesh/2024	79	Threonine	Isoleucine
	PQ807429/Bangladesh/2024	101	Serine	Glycine
	PQ807429/Bangladesh/2024	122	Proline	Serine
	PQ807429/Bangladesh/2024	206	Histidine	Arginine
O	PQ801122/Bangladesh/2024	13	Alanine	Threonine
	PQ807430/Bangladesh/2024	13	Alanine	Threonine
	PQ801122/Bangladesh/2024	45	Lysine	Glutamine
	PQ801122/Bangladesh/2024	46	Aspartic acid	Asparagine
	PQ801122/Bangladesh/2024	139	Serine	Glycine
	PQ801122/Bangladesh/2024	140	Serine	Alanine
	PQ807430/Bangladesh/2024	142	Threonine	Proline
	PQ807430/Bangladesh/2024	158	Threonine	Proline
	PQ801122/Bangladesh/2024	171	Threonine	Alanine
	PQ801122/Bangladesh/2024	197	Serine	Aspartic acid
	PQ801122/Bangladesh/2024	198	Glutamic acid	Glutamine

**Table 3:** VP1 GH loop region mutations identified in FMDV serotype O sequences.

Strain	Sequence	Position	Majority AA	Buffalo AA
Asia‐1	—	—	—	—
O	PQ801122/Bangladesh/2024	139	Serine	Glycine
	PQ801122/Bangladesh/2024	140	Serine	Alanine
	PQ807430/Bangladesh/2024	142	Threonine	Proline
	PQ807430/Bangladesh/2024	158	Threonine	Proline

### 3.7. Patterns of Selection Pressure in the VP1 Region of Serotype Asia‐1 and O FMDV

Selection pressure analysis identified evidence of both purifying and positive selection acting on codon sites within the VP1 gene of serotype Asia‐1 and O FMDV. Codon sites identified by FEL, SLAC, FUBAR, and MEME provided complementary evidence of selection, enabling the detection of both pervasive and episodic evolutionary processes. Overall, the analyses indicated that the VP1 gene in both serotypes is predominantly shaped by purifying selection, reflecting strong evolutionary constraints maintaining the structural and functional stability of the protein. In contrast, signals of positive selection were detected at comparatively fewer codon sites in both serotypes (Table S4). In serotype Asia‐1 sequences, overlapping sites identified by multiple methods suggested localized adaptive changes, while MEME detected additional sites under episodic diversifying selection. Similarly, in serotype O sequences, a limited number of codon sites were identified under positive selection, with several being supported by more than one analytical method. Overall, these findings indicate that selection pressure across the VP1 gene is unevenly distributed, with purifying selection dominating most regions and only restricted sites showing evidence of adaptive evolution.

### 3.8. Genetic Diversity, Neutrality, and Haplotype Network Analysis of the VP1 Gene

**Figure 5:**
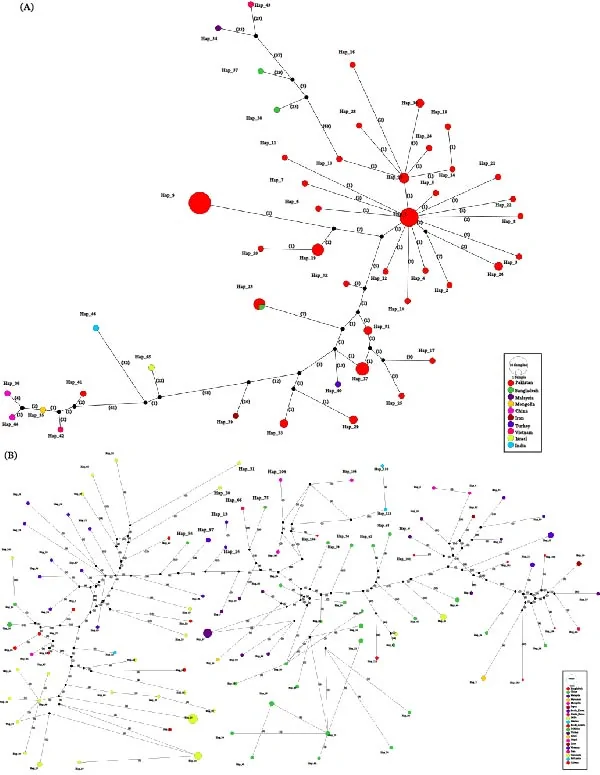
Haplotype network of FMDV serotypes. (A) Asia‐1 FMDV VP1 sequences showing genetic relationships among haplotypes from Bangladesh and other Asian countries. Circle size represents haplotype frequency, colors indicate countries, and numbers on branches represent mutational steps. (B) Type O FMDV VP1 sequences showing genetic relationships among haplotypes from Bangladesh and other countries. Circle size represents haplotype frequency, colors indicate countries, and numbers on branches represent mutational steps.

Genetic diversity analysis of the VP1 gene revealed considerable nucleotide variation among both serotype Asia‐1 and O FMDV sequences, suggesting genetically diverse circulating viral populations. The Asia‐1 dataset showed moderate nucleotide diversity (*π* = 0.05258) and high haplotype diversity (Hd = 0.954). Neutrality analysis produced negative Tajima’s *D* and Fu and Li’s 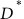 and 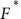 values (*p*  > 0.05), suggesting no significant deviation from neutral evolution (Table 4). The haplotype network showed that Bangladeshi Asia‐1 haplotypes were shared with other Asian populations and were mostly separated by a few mutational steps, with occasional divergent lineages (Figure 5A). In comparison, the serotype O dataset exhibited higher genetic diversity (*π* = 0.11642 and Hd = 0.993), reflecting greater viral diversification. Neutrality tests similarly showed negative but non‐significant values (*p*  > 0.05), indicating largely neutral evolution with the influence of purifying selection (Table 4). The haplotype network showed shared haplotypes between Bangladesh and other Asian countries, with most haplotypes connected by small mutational differences and a few divergent lineages (Figure 5B). Overall, serotype O showed greater variability than Asia‐1, while both serotypes maintained functional constraints within the VP1 region.

**Table 4:** Genetic diversity and neutrality test statistics of the VP1 gene in FMDV serotypes Asia‐1 and O.

Parameter	Asia‐1	O
Number of sequences (*n*)	85	144
Analyzed sites	625	627
Polymorphic sites (*S*)	247	321
Nucleotide diversity (*π*)	0.05258	0.11642
Number of haplotypes (*h*)	46	112
Haplotype diversity (Hd)	0.954	0.993
Tajima’s *D*	−1.624 (0.10 > *p* > 0.05)	−0.522 (*p* > 0.10)
Fu and Li’s *D* ^∗^	−1.316 (*p* > 0.10)	−0.356 (*p* > 0.10)
Fu and Li’s *F* ^∗^	−1.737 (*p* > 0.10)	−0.519 (*p* > 0.10)

^∗^signifies that the test is designed for sampled sequences data without an out‐group, specifically contrasting the number of singletons (variants present in only one individual) with the total number of mutations or pairwise differences.

## 4. Discussion

This study provides the first comprehensive molecular epidemiological investigation of FMDV in buffalo populations of Bangladesh, revealing high individual‐level (41.0%) and herd‐level (88.1%) prevalence. Such widespread occurrence indicates active viral circulation within the buffalo production systems and suggests that buffaloes are frequently exposed to FMDV under endemic conditions. The high herd‐level prevalence may be explained by common management practices in Bangladesh, including communal grazing, unrestricted animal movement, shared water sources, and close contact between the buffalo and cattle. These conditions facilitate repeated viral introduction and sustained transmission within and among herds. Our findings are consistent with previous reports from Bangladesh and other endemic regions where FMDV prevalence was strongly associated with mixed‐species husbandry systems and high animal contact rates [1, 3, 35]. Recent epidemiological analyses have further demonstrated that recurrent FMD outbreaks in Bangladesh are driven by livestock movement networks and the persistence of endemic viral reservoirs [3, 22]. Therefore, the high prevalence observed in the present study likely reflects both local transmission within buffalo herds and continuous exposure through inter‐herd and interspecies interactions.

The co‐circulation of serotypes O and Asia‐1 observed in buffalo populations mirrors the epidemiological situation previously reported in cattle populations of Bangladesh and neighboring South Asian countries [3, 18, 36]. Serotype O remains the dominant serotype across South Asia because of its broad host range, efficient transmission dynamics, and continued evolutionary adaptation, whereas Asia‐1 persists at lower frequencies through localized transmission cycles. The detection of identical serotypes in buffalo and cattle supports the hypothesis that both species participate in interconnected transmission networks. In Bangladesh, buffalo and cattle frequently share grazing fields, water bodies, and livestock markets, creating ecological interfaces that facilitate cross‐species transmission. Similar transmission dynamics have recently been reported in Kenya, Pakistan, and India, where buffalo populations contributed to the maintenance and dissemination of FMDV within mixed livestock ecosystems [8, 15, 36]. These findings emphasize the importance of continuous molecular surveillance and the use of polyvalent vaccines that protect multiple circulating serotypes, which can save animals and reduce economic losses [5, 13].

Phylogenetic analysis demonstrated that buffalo‐derived isolates clustered closely with contemporary cattle isolates, suggesting possible interspecies transmission; however, this interpretation should be considered cautiously given the limited number of sequenced isolates. This close genetic relationship suggests frequent viral exchange among livestock populations rather than independent evolution within the buffalo. Such patterns are expected in endemic settings where animal movement, livestock trading, and transboundary animal exchange facilitate the dissemination of genetically related viral strains. Recent genomic studies have shown that FMDV circulation in South Asia is characterized by continuous lineage replacement and regional connectivity, particularly among countries sharing porous borders and extensive livestock movement networks [18, 22, 37, 38]. Notably, the identification of the ME‐SA/Ind2001e lineage for serotype O and South Asian lineages for Asia‐1 indicates ongoing endemic circulation rather than the emergence of novel evolutionary groups. This observation strengthens previous evidence that dominant regional lineages continue to drive FMD epidemiology in Bangladesh [3, 14, 39, 40].

The observed VP1 variability and circulation of multiple serotypes may have important implications for vaccine effectiveness in Bangladesh. The amino acid substitutions identified within antigenically important regions of VP1, particularly near the GH loop, may have important implications for vaccine performance. The GH loop contains major neutralizing epitopes, responsible for host antibody recognition and receptor binding. Mutations within this region can alter the antigenic structure, potentially reducing cross‐protective immunity induced by existing vaccine strains. Although the overall VP1 sequence remained highly conserved, the observed substitutions suggest ongoing immune‐mediated viral evolution under endemic conditions. Similar patterns have recently been reported in Bangladesh and India, where antigenic variation within VP1 was associated with the emergence of genetically distinct field strains and concerns regarding vaccine matching [3, 14, 36]. These findings reinforce the importance of regular vaccine‐strain matching and continuous molecular surveillance to maintain vaccine effectiveness.

The observed relationship between genetic and geographic distance is consistent with previously described isolation‐by‐ distance patterns for FMDV and other RNA viruses. In such systems, geographically closer populations are more likely to exchange viral strains due to localized transmission. Earlier molecular epidemiological analyses have demonstrated that spatial proximity often predicts genetic similarity among FMDV isolates, reflecting localized transmission dynamics interspersed with occasional long‐distance dissemination events [41, 42]. The moderate Mantel correlation detected here supports the hypothesis that geographic structure plays an important role in shaping viral diversity in endemic livestock systems [37]. The positive relationship between genetic and temporal distance suggests a gradual accumulation of genetic variation over time; however, this finding should be interpreted with caution.

Selection pressure analysis further demonstrated that the VP1 gene is predominantly shaped by purifying selection, with only a limited number of codon sites showing evidence of positive or episodic diversifying selection. This evolutionary pattern is expected because VP1 plays essential structural and functional roles in receptor binding, capsid assembly, and viral infectivity. Consequently, strong functional constraints limit the accumulation of mutations across most codon sites. Similar evolutionary patterns have been reported in recent FMDV studies from South Asia and the Middle East, where purifying selection predominated, while adaptive evolution remained restricted to antigenically important regions [14, 37].

Population genetic analysis revealed high haplotype diversity in both serotypes, with greater nucleotide diversity observed in serotype O compared with Asia‐1. Similar patterns have been reported in previous studies from South Asia and the Middle East [38, 43, 44]. This may be attributed to the wider host range and transmission dynamics of serotype O compared to those of Asia‐1, although broader sampling would be required to confirm this pattern. The negative neutrality statistics observed in this study, although not statistically significant, are consistent with previous reports indicating population expansion or weak purifying selection rather than strong directional selection [14]. These patterns may indicate the gradual accumulation of genetic variation within circulating FMDV populations.

In this study, buffalo showed high herd‐level FMDV prevalence and carried both serotypes O and Asia‐1. Their close genetic relationship with regional strains suggests shared transmission networks and possible interspecies spread. In Bangladesh, communal grazing and mixed‐species rearing likely facilitate continuous FMDV transmission among livestock populations. These findings suggest that buffalo may have a role beyond incidental infection and could contribute to the local maintenance and amplification of FMDV transmission, particularly under conditions of high animal density and unrestricted movement. Thus, buffalo may act as maintenance hosts or amplifiers of FMDV in endemic conditions; however, further longitudinal studies are necessary to distinguish between maintenance and spillover dynamics.

A limitation of this study is that sequencing was performed on a limited number of isolates selected primarily based on the amplification quality and geographic distribution. This approach did not fully account for herd‐level representation, epidemiological clustering, or temporal variation, which may influence phylogenetic interpretation, and lineage‐specific prevalence could not be determined. Consequently, the findings provide preliminary insights into circulating FMDV lineages rather than definitive conclusions regarding transmission dynamics or population structure. Future studies incorporating larger sample sizes, stratified sampling across herds, and longitudinal designs will be essential to better understand FMDV transmission patterns in the buffalo population.

## 5. Conclusion

This study provides baseline molecular epidemiological insights into FMDV in buffalo populations of Bangladesh, revealing high individual‐ and herd‐level prevalence and indicating widespread endemic circulation. The co‐circulation of serotypes O and Asia‐1, clustering within established South Asian lineages, suggests ongoing local transmission and regional connectivity. Despite the overall conservation of genomic regions such as VP1, localized amino acid variations in antigenically important sites indicate potential viral evolution, although this requires further investigation. These findings highlight the buffalo as an important yet underrecognized component of FMD epidemiology in Bangladesh and underscore the need to integrate the buffalo into national surveillance, vaccination, and control strategies.

## Author Contributions

**Abdullah Mohammad Asif**: data curation, formal analysis, methodology, investigation, writing – original draft, writing – review and editing. **Tajul Islam Mamun**: data curation, formal analysis, investigation, software, writing – original draft, writing – review and editing. **Md. Rokibul Hasan Shanto**: data curation, investigation, validation, writing – original draft, writing – review and editing. **Md. Khademul Islam**: data curation, formal analysis, writing – original draft, writing – review and editing. **Md. Mashfiq Hossain Khadem**: data curation, investigation, methodology, writing – original draft, writing – review and editing. **Uttama Acharjee**: data curation, writing – original draft, writing – review and editing. **Md. Shazzadul Kabir and Asikur Rahman**: data curation, investigation, writing – original draft, writing – review and editing. **Md. Shakil Mahmud Supto**: data curation, visualization, writing – original draft, writing – review and editing. **Syed Muktadir Al Sium**: data curation, formal analysis, software, writing – original draft, writing – review and editing. **Amam Zonaed Siddiki**: data curation, validation, writing – original draft, Writing – review and editing. **Md Bashir Uddin**: conceptualization, formal analysis, investigation, methodology, funding acquisition, project administration, supervision, writing – original draft, writing – review and editing. **Syed Sayeem Uddin Ahmed**: conceptualization, formal analysis, funding acquisition, investigation, methodology, project administration, supervision, software, validation, writing – original draft, writing – review and editing.

## Funding

The research grant was provided by the Bangladesh Livestock Research Institute, Bangladesh (BLRI) (Grant #21‐837).

## Conflicts of Interest

The authors declare no conflicts of inerest.

## Supporting Information

Additional supporting information can be found online in the Supporting Information section.

## Supporting information


**Supporting Information** Figure S1: Maximum likelihood phylogenetic tree based on partial VP1 nucleotide sequences of FMDV (A) serotype Asia‐1 and (B) serotype O including global reference sequences. Figure S2: Maximum‐likelihood phylogenetic tree showing the evolutionary relationship of FMDV sequences generated in this study with Asian reference genomes. The tree was reconstructed using three whole‐genome sequences and eight partial sequences generated in this study, together with 509 publicly available FMDV reference genomes retrieved from GenBank. Sequences were aligned using MAFFT v7, and phylogenetic inference was performed using RAxML v8 under the GTR substitution model. Background clade colors indicate FMDV serotypes, while the outer color strip denotes the country of isolation for reference sequences where metadata were available. The newly generated sequences from this study are included in the tree without country‐color annotation. The tree was visualized and annotated using iTOL v7. Table S1: primers used for universal serotyping of FMDV by RT‐PCR. Table S2: BLAST analysis of nucleotide sequences of FMDV isolates obtained in the present study. Table S3: Mantel test results for genetic, geographic, and temporal distance comparisons among analyzed isolates. Table S4: amino acid sites under selection pressure across the whole FMDV phylogeny. Sites detected with multiple methods are in bold; symbol “–” indicates that no site was identified for a gene using this method.

## Data Availability

The datasets supporting the conclusions of this article are included within the article and supporting files. The sequence data generated in this study have been partially deposited in the NCBI GenBank database. The whole‐genome sequences (*n* = 3) are available under accession numbers PQ807429.1, PQ807430.1, and PQ801122.1 and partial sequences (*n* = 4) under accession numbers PQ492269.1, PQ634872.1, PQ634966.1, and PQ613602.1. The remaining partial sequences are currently under submission and will be made publicly available upon acceptance.
