# Supplementary material for: Endemic Circulation and Genetic Characterization of Foot‐and‐Mouth Disease Virus in Buffalo Populations of Bangladesh
Source: Transbound Emerg Dis. 2026 Sep 12;2026:3107202. doi: 10.1155/tbed/3107202 (PMC13570446; doi:10.1155/tbed/3107202)
Supplement: Supplementary file 1 [file TBED-2026-3107202-s001.zip › Supplementary table - Revised_CLEAN.docx]

Supplementary Table S1: Primers used for universal serotyping of FMDV by RT-PCR

| **Primer**  **Name** | **Serotype** | **Sequences (5’-3’)** | **Gene** | **PCR Products (bp)** | **Reference** |
| --- | --- | --- | --- | --- | --- |
| NPP-F1 | Universal | GAGTCCAACCCTGGGCCCTTC | Polyprotein | 494 | This study |
| NPP-R1 |  | ATGTCACGTGCTTTGAGCTG |  |  |  |
| RF2-F | Universal | CCTACCTCCTTCAACTACGG | Polyprotein | 215 | El-Kholy et al., 2007 |
| RF2-R |  | GAAGGGCCCAGGGTTGGACTC |  |  |  |

Supplementary Table S2: BLAST analysis of nucleotide sequences of FMDV isolates obtained in the present study

| **Sample No.** | **Study area** | **Species** | **Strain** |
| --- | --- | --- | --- |
| BD S1 | Noakhali | Buffalo | O |
| BD S2 | Rajshahi | Buffalo | O |
| BD S3 | Rajshahi | Buffalo | O |
| BD S4 | Rajshahi | Buffalo | Asia 1 |
| BD S5 | Sylhet | Buffalo | O |
| BD S6 | Sylhet | Buffalo | Asia 1 |
| BD S7 | Noakhali | Buffalo | O |
| BD S8 | Noakhali | Buffalo | Asia 1 |

Supplementary Table S3: Mantel test results for genetic, geographic, and temporal distance comparisons among analyzed isolates.

| **Statistic** | **Value** | **Significance (P-value)** | **Method** | **Permutations** |
| --- | --- | --- | --- | --- |
| Mantel Correlation (Genetic vs Geographic) | 0.4792 | 0.001 | Pearson | 999 |
| Mantel Correlation (Genetic vs Temporal) | 0.4792 | 0.001 | Pearson | 999 |

Supplementary Table S4: Amino acid sites under selection pressure across the whole FMDV phylogeny. Sites detected with multiple methods are in bold; symbol ‘–’ indicates that no site was identified for a gene using this method.

| **Strain** | Amino Acid/Codon sites | | | |
| --- | --- | --- | --- | --- |
|  | **EFL** | **FUBAR** | **MEME** | **SLAC** |
| Asia-1 | **84** | **84**,**207** | 48,52,92,133,184,**207** | - |
| O | **47,96** | **133** | 9,45,**47**,83,**96**,**133**,141,144,197 | - |
